# Single Catheter Use as the Default Approach for Coronary Angiography and Intervention in Patients with ST-Elevation Myocardial Infarction

**DOI:** 10.3390/diagnostics16132049

**Published:** 2026-06-30

**Authors:** Yusuf Can, Ömer Faruk Erkan, Muhammet Taşdemir, Mustafa Şahinöz, Ahmet Can Çakmak, Fahrettin Turna, Ali Baş, Mehmet Şirin Yıldız, Nimet Uçaroğlu Can, Lulieta Kurani Allaraj, Havva Kocayiğit, İbrahim Kocayiğit

**Affiliations:** 1 Department of Cardiology, Faculty of Medicine, Sakarya University, Adapazari 54188, Turkey; 2 Department of Cardiology, Sakarya Training and Research Hospital, Adapazari 54100, Turkey; omerfarukerkan@hotmail.com.tr (Ö.F.E.); drahmetcancakmak@gmail.com (A.C.Ç.); fturna_53@hotmail.com (F.T.); dralibas43@gmail.com (A.B.); mehmet_sirin65@hotmail.com (M.Ş.Y.); flowersaallaraj@gmail.com (L.K.A.); ikocayigit@gmail.com (İ.K.); 3 Department of Cardiology, Yenikent State Hospital, Sakarya 54290, Turkey; muhammet.75256@gmail.com; 4 Department of Cardiology, Sadıka Sabancı State Hospital, Sakarya 54580, Turkey; mustafa-945@hotmail.com; 5 Department of Neurology, Sakarya Training and Research Hospital, Adapazari 54100, Turkey; nimetucaroglu37@hotmail.com; 6 Department of Anesthesiology and Reanimation, Sakarya Training and Research Hospital, Adapazari 54100, Turkey; havvakocayigit@gmail.com

**Keywords:** transradial access, single-catheter technique, percutaneous coronary intervention

## Abstract

**Background/Objectives**: Transradial access (TRA) is a standard approach in the management of ST-segment elevation myocardial infarction (STEMI); however, evidence on using a single catheter for both diagnostic angiography and percutaneous coronary intervention (PCI) is limited. This study evaluates the feasibility and clinical outcomes of using a single Judkins Left (JL) 3.5 guiding catheter via TRA in STEMI patients. **Methods**: A total of 1139 patients undergoing radial access PCI with a single JL 3.5 catheter were included. Procedural success was defined as completing both diagnostic coronary angiography and PCI without catheter exchange. Procedural characteristics and access-site complications were evaluated. **Results**: The success rate of completing diagnostic angiography and PCI using a single JL 3.5 guiding catheter without catheter exchange was 91.1%. Compared to procedures requiring multiple catheters, the single-catheter group had significantly lower total contrast volume (200 vs. 250 mL), procedure time (20 vs. 30 min), fluoroscopy time (10.3 vs. 17.6 min), radiation dose (358 vs. 545 mGy), and needle-to-balloon time (6 vs. 9 min), all with *p* < 0.001. Access-site complications were also lower (8.2% vs. 15.8%; *p* = 0.010), primarily due to reduced radial artery spasm (4.0% vs. 12.9%; *p* = 0.001). **Conclusions**: A single JL 3.5 catheter strategy via transradial access is a safe, efficient, and effective method for STEMI intervention, offering significant procedural and clinical advantages.

## 1. Introduction

Primary percutaneous coronary intervention (pPCI) via the radial artery has become the first-line reperfusion strategy for patients with ST-segment elevation myocardial infarction (STEMI). Cardiac catheterization with transradial arterial support has been found to be associated with a lower risk of complications (especially intervention site complications), better patient comfort, shorter patient recovery time, and lower costs [1,2,3,4,5].

Radial artery intervention complications, such as radial spasm, rupture, pseudoaneurysm, a-v fistula, and bleeding, may be observed due to reasons such as the radial artery being smaller in diameter, having congenital abnormalities, and being tortuous. In addition, embolism and vascular complications may increase due to atherosclerotic plaques and tortuosity in the brachial, axillary, subclavian, and brachiocephalic arteries [6]. Repeated catheter use via the radial artery may result in increased radial spasm, radial artery occlusion, bleeding, embolic complications, and radiation exposure. For this reason, a single Tiger catheter is routinely used to image both the right and left coronary arteries in diagnostic coronary angiography [7]. The 6-French Ikari Left guiding catheter has been used for both diagnostic coronary angiography and coronary interventional procedures [8,9], while the 6-French Judkins Left (JL) 3.5 guiding catheter has been used for diagnostic coronary angiography of the right and left coronary arteries [10,11]. In a small number of patients, the JL 3.5 guiding catheter was used for both diagnostic coronary angiography and interventional procedures [12]. This study evaluated the feasibility and reliability of imaging and treatment of right and left coronary systems using right radial access and a single JL 3.5 guiding catheter in patients presenting with acute STEMI.

## 2. Methods

### 2.1. Study Population

The study population consisted of STEMI patients who were scheduled to undergo coronary intervention through the right radial artery with a single catheter between May 2022 and September 2023. All 1139 patients, regardless of STEMI type, were included in the study.

STEMI was defined based on the following criteria: (i) chest pain lasting more than 30 min within 12 h of presentation; (ii) ECG findings of ST-segment elevation measured from the J point in at least two contiguous leads, with ≥2.5 mm in men younger than 40 years, ≥2 mm in men older than 40 years, ≥1.5 mm in women in leads V2–3, and ≥1 mm in other leads or the presence of a new bundle branch block pattern; and (iii) ST-segment depression in leads V1–3 with terminal positive T waves, which is indicative of myocardial ischemia and should also prompt consideration of ≥0.5 mm ST-segment elevation in leads V7–9 for diagnosis [5,13].

Hypertension was defined as a documented diastolic blood pressure of 90 mmHg or higher and/or a systolic blood pressure of 140 mmHg or higher in at least two measurements or current use of antihypertensive medication. Diabetes mellitus was defined by a glucose level greater than 200 mg/dL, a glycated haemoglobin level greater than 6.5% on any test, a fasting plasma glucose level greater than 126 mg/dL, or current use of antidiabetic medication. Dyslipidemia was defined based on previously established criteria [14]. Smokers were identified as individuals who were smoking regularly within the last six months [14].

Exclusion criteria included individuals under 18 years of age; patients diagnosed with malignancy or undergoing cancer treatment; individuals presenting with pulmonary edema, cardiogenic shock, decompensated heart failure, sudden cardiac death, chronic renal failure (GFR < 15), upper extremity artery disease, arteriovenous fistula, upper extremity deformities, or with a history of coronary artery bypass surgery; and pregnancy. The study was approved by the Ethics Committee of Sakarya University Faculty of Medicine (Ethics Committee Number: E-71522473-050.04-372969, approval date 27 June 2024).

### 2.2. Procedure

In patients whose right radial artery could be palpated, after local anaesthetic was applied, a 7-cm hydrophilic-coated 6F sheath was inserted with the assistance of a 21 G needle and a 40 cm 0.018 guidewire, and then 200 mcg nitroglycerin and 5000 IU unfractionated heparin were administered intraarterially. A JL 3.5 guiding catheter was employed for both right and left coronary angiography and coronary intervention.

**Figure 1:**
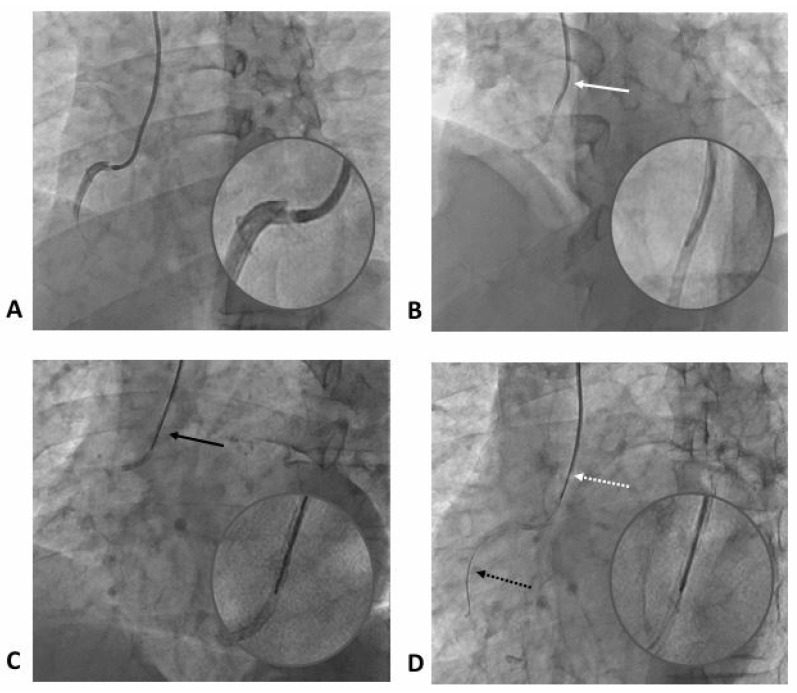
Fluoroscopic demonstration of different guidewire-assisted catheter configurations used during the procedure. (**A**) Catheter position without guidewire assistance. (**B**) The white arrow indicates the soft tip of the 0.038-inch guidewire. (**C**) The black arrow indicates the rigid tip of the 0.038-inch guidewire. (**D**) The white dashed arrow indicates the rigid tip of the 0.038-inch guidewire, while the black dashed arrow indicates the 0.014-inch guidewire used for additional support.

The left main coronary artery was first engaged with a JL 3.5 guiding catheter, and imaging was carried out. If a culprit lesion was identified in the left main coronary artery, left anterior descending artery, left circumflex, ramus intermedius artery, or their branches, the procedure continued. After imaging or intervention of the left coronary system, the JL 3.5 guiding catheter was inserted into the right coronary ostium by clockwise rotation. If this failed, a 0.038-inch guidewire was used to aid the manipulation. The right coronary artery was visualized using a JL 3.5 guiding catheter, and coronary intervention was performed with the same catheter in the presence of a culprit lesion. If this also failed, in cases of subclavian artery tortuosity, right coronary outlet anomaly, or insufficient guiding catheter support, the rigid tip of the 0.038-inch guidewire was advanced into the catheter to facilitate manipulation of the JL 3.5 guiding catheter. The rigid tip of the 0.038-inch guidewire was advanced to a point 5–20 mm from the distal orifice of the catheter, and the rigid tip was kept within the catheter. The primary curve of the catheter was rectified, such that the morphology of the JL 3.5 resembled that of a JR 4, and selective catheterization of the right coronary artery was performed. This approach allowed for easy catheter manipulation. Representative fluoroscopic images demonstrating the guidewire-assisted reshaping technique of the JL 3.5 guiding catheter are shown in Figure 1. If the whole process failed, in situations with anomalous right coronary ostium, subclavian tortuosity, or insufficient guiding catheter support, the 0.014-inch guidewire was advanced to the distal right coronary artery alongside the rigid tip of the 0.038-inch guidewire. The 0.038-inch guidewire was then pulled back to proceed with coronary imaging and intervention. Procedural success with the single-catheter strategy was defined as successful completion of the entire procedure, including diagnostic coronary angiography, culprit vessel cannulation, and percutaneous coronary intervention when indicated, using only the JL 3.5 guiding catheter without the need for catheter exchange. Cases requiring conversion to another guiding catheter or crossover to femoral access were classified as unsuccessful single-catheter procedures.

Recorded data included radiation exposure, fluoroscopy time, contrast volume used, total procedure duration, types and numbers of catheters used, the number of stents inserted, pre-dilation and post-dilation counts, puncture duration, and the vessel that underwent treatment. Radial artery spasm (RAS) is clinically defined as a sudden narrowing of the radial artery, which can interfere with catheter-based procedures. The diagnosis is based on a questionnaire assessing five signs: ongoing forearm pain, pain triggered by catheter movement, pain when removing the introducer, difficulty manoeuvring the catheter due to spasm, and significant resistance encountered during introducer withdrawal. RAS is clinically diagnosed if two or more of these signs are detected [15]. Assessment of radial artery spasm was performed prospectively during the procedure by the operating interventional cardiologist using a standardized institutional assessment form. To reduce subjectivity, radial artery spasm was not diagnosed based on a single symptom or operator impression but required the presence of at least two predefined clinical criteria. All participating operators were experienced in transradial interventions and routinely applied the same assessment criteria throughout the study period. Haemostasis was established at the radial puncture site using a compression band following the procedure. The compression was gradually decreased two hours post-procedure, and the band was removed after confirming bleeding control. Coronary artery disease was defined as 50% or greater stenosis in the coronary arteries. These procedures were carried out by various operators skilled in radial angiography.

### 2.3. Statistical Analysis

Statistical analyses were carried out using the SPSS software package, version 27.0 (IBM Corp., Armonk, NY, USA). Categorical (qualitative) data were summarized as frequencies and percentages. Numerical (quantitative) data were expressed as mean ± standard deviation. The Kolmogorov–Smirnov test was employed to check whether continuous variables followed a normal distribution. The Pearson chi-square test was applied to compare categorical variables, with results reported in numbers and percentages. For continuous variables with a normal distribution, an independent samples t-test was used, and findings were expressed as mean ± standard deviation. For variables that did not follow a normal distribution, the Mann–Whitney U test was performed, with results shown as median and interquartile range. Univariate logistic regression analysis was initially used to identify factors associated with successful completion of the procedure using a single catheter strategy. Variables found to be statistically significant in the univariate analysis were subsequently included in a multivariate logistic regression model.

## 3. Results

The study included 1139 patients diagnosed with STEMI who underwent single catheter coronary intervention via the radial artery. Of these patients, 909 (79.8%) were male and 230 (20.2%) were female. The mean age of the patients was 60 ± 11 years. The success rate of performing interventions on both the left and right coronary arteries using a single JL 3.5 catheter was as high as 91.1%. Patients in whom the procedure was completed with a single JL 3.5 catheter were compared with patients who required multiple catheters. Patients treated with a single JL 3.5 guiding catheter for percutaneous coronary intervention were younger and predominantly male compared to those who required multiple catheters (59.6 ± 11.5 years vs. 62 ± 12.8 years; *p* = 0.046 and 80.5% vs. *p* = 0.048 and 72.3%, respectively). Patients who underwent percutaneous coronary intervention with a single JL 3.5 guiding catheter had a lower prevalence of history of coronary artery disease and fewer cases of inferior myocardial infarction compared to those treated with multiple catheters (15.6% vs. 29.7%; *p* < 0.001 and 55.7% vs. 66.3%; *p* = 0.016, respectively). The other clinical and demographic characteristics of patients who underwent percutaneous coronary intervention with a JL 3.5 guiding catheter and those treated with multiple catheters were similar and are presented in Table 1.

The number and duration of punctures were similar in both groups of patients undergoing percutaneous coronary intervention using a single JL 3.5 guiding catheter compared to patients treated with more than one catheter (1.3 ± 0.6 vs. 1.4 ± 0.7; *p* = 0.091 and 17.6 ± 14.7 vs. 18.5 ± 17.1; *p* = 0.554, respectively). The total contrast volume (mL) (200 vs. 250; *p* < 0.001), procedure time (min) (20 vs. 30; *p* < 0.001), fluoroscopy time (min) (10.3 vs. 17.6; *p* < 0.001), needle-to-balloon time (min) (6 vs. 9; *p* < 0.001), and total radiation dose (mGy) (358 vs. 545; *p* < 0.001) were significantly lower in patients treated with percutaneous coronary intervention using a JL 3.5 guiding catheter compared to those treated with multiple catheters. Although the median door-to-balloon time was identical between groups (15 vs. 15 min), the overall distribution differed significantly, with a narrower interquartile range in the single-catheter group (12–16 vs. 15–18), resulting in a statistically significant difference on non-parametric comparison (*p* < 0.001). The frequency of balloon pre-dilatation, balloon post-dilatation, and use of thrombus aspiration was comparable between the two groups (94.9% vs. 96%, *p* = 0.946; 56% vs. 52.5%, *p* = 0.499; and 3.6% vs. 5.0%, *p* = 0.480, respectively).

Femoral artery access was used in 24 patients in whom percutaneous coronary intervention via the radial artery was not successful. Patients who underwent percutaneous coronary intervention with a JL 3.5 guiding catheter had fewer coronary artery anomalies and a similar number of diseased vessels compared to those treated with multiple catheters (0.4% vs. 4.0%; *p* = 0.003 and 1.4 ± 0.6% vs. 1.4 ± 0.6%; *p* = 0.886, respectively). Balloon-only angioplasty was performed less frequently in patients undergoing percutaneous coronary intervention with a JL 3.5 guiding catheter compared with those treated with multiple catheters, although Drug-eluting stent use was more common (4.5% vs. 11.9%; *p* = 0.001 and 94.4% vs. 86.1%; *p* = 0.001, respectively). In patients in whom percutaneous coronary intervention was completed with a JL 3.5 guiding catheter, the soft tip of the 0.038 wire, the rigid tip of the 0.038 wire, and the 0.038 rigid tip together with the 0.014 wire were used more frequently. The number of stents used was higher in patients in whom percutaneous coronary intervention was completed with a JL 3.5 guiding catheter.

Of the culprit lesions, 41.7% were located in the right coronary artery, 38.1% in the left anterior descending artery, 18.7% in the left circumflex artery, 0.4% in the left main coronary artery, and 1.1% in the ramus intermedius. The procedure was completed successfully in 90% of the patients with a JL 3.5 guiding catheter inserted via the radial artery. In patients who underwent percutaneous coronary intervention with a JL 3.5 guiding catheter, the culprit lesion was predominantly in the left coronary system, whereas in patients treated with multiple catheters, the culprit lesion was mostly in the right coronary artery (59.3% vs. 47.5%; *p* = 0.001 and 40.7% vs. 52.5%; *p* = 0.001, respectively). Table 2 presents the procedural features of the study population.

**Figure 2:**
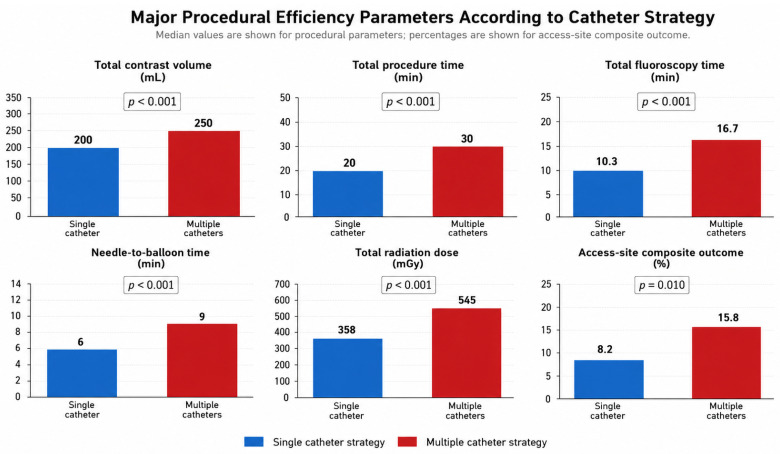
Comparison of major procedural efficiency parameters between single-catheter and multiple-catheter strategies.

Patients treated with a JL 3.5 guiding catheter for percutaneous coronary intervention had a lower rate of access-site composite outcomes compared to those who required multiple catheters (8.2% vs. 15.8%; *p* = 0.010). The main reason for the lower access site composite results in patients who underwent percutaneous coronary intervention with a JL 3.5 guiding catheter compared to those who required more than one catheter was the lower incidence of radial artery spasm (4.0% vs. 12.9%; *p* = 0.001). The 30-day reinfarction, stroke, and mortality rates were comparable between patients who underwent percutaneous coronary intervention with a single catheter and those who required multiple catheters (1.8% vs. 1.0%, *p* = 0.458; 0.2% vs. 0%, *p* = 0.830; and 1.8% vs. 2.0%, *p* = 0.569, respectively). Iatrogenic coronary dissection occurred in three patients during right coronary artery intervention. It was statistically more frequent in the group treated with multiple catheters than in the single-catheter group (2.0% vs. 0.1%; *p* = 0.022). Complications and 30-day outcomes are presented in Table 2, while the overall comparison of major procedural efficiency parameters is graphically summarized in Figure 2.

**Figure 3:**
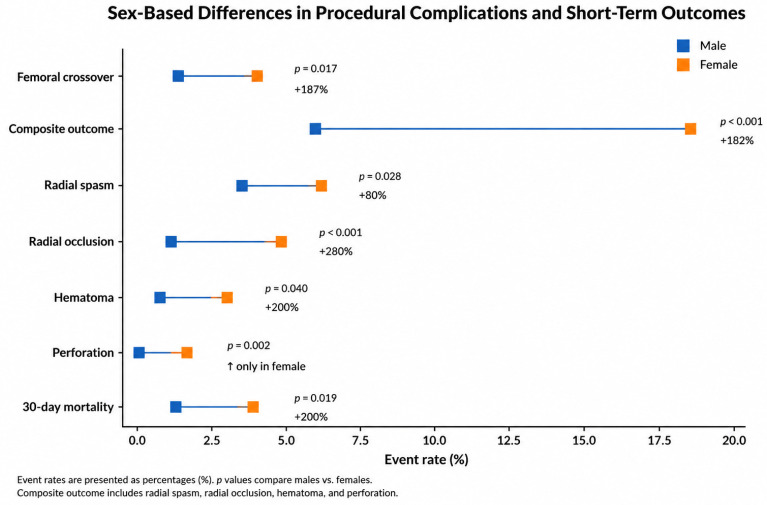
Sex-based differences in procedural complications and short-term outcomes.

A sex-based subgroup analysis was performed to further evaluate procedural complications and short-term outcomes. Female patients had significantly higher rates of femoral crossover, access-site complications, radial artery spasm, radial artery occlusion, hematoma formation, arterial perforation, and 30-day mortality compared with male patients. These findings are summarized in Table 3, while the relative differences in major sex-related procedural complications and short-term clinical outcomes are visually illustrated in Figure 3.

Values are presented as number (percentage). Access-site composite outcome includes radial artery spasm, radial artery occlusion, hematoma, and arterial perforation.

Parameters predicting successful completion of the procedure using a single catheter in STEMI patients were evaluated by logistic regression analysis (Table 4). For this purpose, age, male gender, a history of coronary artery disease, and the presence of a coronary artery anomaly were analysed in terms of whether they were associated with completion of the procedure with a single catheter. Age, male gender, a history of coronary artery disease, the number of stents, and coronary artery anomalies, all risk factors that were found to be correlated with the use of a single catheter as a result of the univariate logistic regression analysis, were also analysed using multivariate logistic regression analysis. In multivariable logistic regression analysis, a history of coronary artery disease (OR 0.44, *p* = 0.001) and the presence of coronary artery anomalies (OR 0.08, *p* = 0.001) were independent negative predictors of successful completion of the procedure with a single catheter. Male sex was associated with a trend toward higher success but did not remain statistically significant after adjustment (OR 1.55, *p* = 0.081).

A radial sheath was inserted in 99.2% of patients. In 2% of cases, the left 3.5 JL guide catheter could not be successfully advanced into the ascending aorta. The most common reason for this failure was radial artery spasm. Although the coronary anatomy was visualized by coronary angiography in 6.9% of patients, percutaneous coronary intervention could not be completed due to a lack of guiding catheter support. The most common reasons for failure to complete percutaneous coronary intervention due to a lack of guiding catheter support were inferior coronary artery take-off and the presence of calcific/long coronary lesions. The mechanisms and causes of failure of transradial percutaneous coronary intervention via radial access with a single catheter are shown in Table 5.

## 4. Discussion

The key findings of our study are as follows: (i) all coronary arteries can be easily, safely, and successfully visualized using a single 3.5 JL guiding catheter through the right radial artery; (ii) coronary interventions can be effectively and safely performed on both the right and left coronary arteries via the right radial artery with the same JL 3.5 guiding catheter; (iii) either the soft or hard tip of the 0.038 guidewire can be used safely in the right coronary artery; (iv) both the 0.014 wire and 0.038 wire were used together to engage the right coronary artery in some cases; (v) contrast volume, procedure time, fluoroscopy time, door-to-balloon time, needle-to-balloon time, and total radiation dose are lower when using the JL 3.5 guiding catheter; and (vi) radial spasm is lower when using the JL 3.5 guiding catheter.

To perform single-catheter imaging of both right and left coronary arteries via the right radial artery, various guiding catheters have been used, including MAC 3.5 (Medtronic, Minneapolis, MN, USA) [12], DxTerity™ TRA (Medtronic, Santa Rosa, CA, USA) [16], Ikari Left 3.5 (Terumo, Tokyo, Japan) [8,9], Tiger II (Terumo, Tokyo, Japan) [7], BLK (Terumo, Somerset, NJ, USA) [17], Kimny (Boston Scientific, Natick, MA, USA) [18], Barbeau (Cordis, Bridgewater, NJ, USA) [19], Jacky (Terumo, Somerset, NJ, USA), and Judkins Left (JL) 3.5 (Cordis, Miami Lakes, FL, USA) [10,11,20]. Ikari Left 3.5 (Terumo, Tokyo, Japan), Kimny (Boston Scientific, Natick, MA, USA), MAC 3.5 (Medtronic, Minneapolis, MN, USA), Judkins Left (JL) 3.5 (Cordis, Miami Lakes, FL, USA), and Q (Q3.5 and Q4 curves, Mach 1 type, Boston Scientific, Hemel Hempstead, UK) guiding catheters have also been used in coronary artery interventions [8,9,12,18,20,21].

Rondan et al. demonstrated that coronary intervention can be performed successfully in both right and left coronary arteries with the JL 3.5 guiding catheter. Rondan et al. showed that the right coronary artery ostium can be successfully engaged using both the soft and rigid tips of the 0.038 guidewire during right coronary artery imaging and interventions via the right radial artery with the JL 3.5 guiding catheter [20]. Erden et al. manually modified the JL 3.5 catheter to create a ‘Jacky-like’ catheter, achieving high procedural success, shorter procedure time, and reduced fluoroscopy time without any complications [11]. Turan et al. showed that, in more than half of the patients in their study, the JL 3.5 catheter allowed for a successful procedure without increasing operative or fluoroscopy times [10]. Similarly, in our study including many STEMI patients, we successfully performed coronary interventions from the right radial artery to the right coronary artery with a JL 3.5 guiding catheter. Similar to previous studies, in our study, contrast volume, procedure time, fluoroscopy time, door-to-balloon time, needle-to-balloon time, and total radiation dose were lower when a single catheter was used.

The most common intervention site complication found in our study was radial spasm, although relatively less radial spasm was found in our study compared to the literature. In our study, the incidence rate of radial spasm was 4.8%, while the published research reports a varying overall rate ranging from 0 to 51% (with a mean incidence of about 5–15%) [22]. The relatively low frequency of RAS may be due to the patient’s focus on chest pain and neglecting the pain at the site of intervention. In addition, the emotional state of the patient at that moment may also be a contributing factor. A further reason for the low incidence of RAS may be explained by the use of a single catheter; in our study, the incidence of radial artery spasm was lower in percutaneous coronary interventions completed with a single catheter.

Although radial artery spasm was more frequent in patients requiring multiple catheters, the direction of this association should be interpreted with caution. In clinical practice, operators generally attempt to complete the procedure with the initially selected catheter despite the occurrence of radial artery spasm, and catheter exchange is more commonly driven by inadequate engagement or insufficient guiding catheter support. Therefore, the higher rate of radial artery spasm observed in the multiple-catheter group may reflect the greater procedural complexity of these cases rather than a direct causal relationship between radial artery spasm and catheter exchange.

Another common complication at the intervention site was radial artery occlusion (RAO). While the rate of RAO was 2.4% in our study, the incidence of RAO in previous studies ranged from 0.8% to 38% (with a mean incidence of about 5–15%) [23,24]. The reason for the relatively low RAO in our study may be the use of dual antiaggregants and the fact that RAO was evaluated at a later stage (in the first month). The use of a single catheter for percutaneous coronary interventions in most patients may account for the relatively low incidence of RAO that we observed.

The main concern in procedures performed on these patients is the risk of catheter-induced iatrogenic coronary artery dissection. Guiding-catheter-induced iatrogenic coronary artery dissection occurs in less than 1% of patients undergoing percutaneous coronary intervention and is associated with deeper penetration of large catheters into smaller, diseased arteries [25,26,27,28]. Data on catheter-induced iatrogenic coronary artery dissection (especially in the right coronary artery) in single catheter studies are limited. Youssef et al. found an incidence rate of RCA dissection of 0.48% in a study using Ikari 3.5 guiding catheters as a single catheter [29]. Compared to interventions using multiple catheters, single-catheter coronary procedures show a lower rate of catheter-induced arterial dissections. We observed this complication in three patients (0.3%); all three patients had a culprit lesion in the right coronary artery, and all were successfully treated with stenting. The rate of catheter-induced coronary artery dissection in our study is similar to previous single-catheter and non-single-catheter studies. Similar to our study, catheter-induced coronary artery dissections are more common in the right coronary artery because more catheter manipulations are performed there.

Catheter crossing can be explained by the following three main mechanisms: (a) a failed radial puncture, (b) the inability to advance a guiding catheter into the ascending aorta, and (c) inadequate guiding catheter support for successful completion of PCI [30]. In a previous study of failed transradial PCI, the most common reason for failure to complete PCI was failure to advance the catheter into the ascending aorta, and the second most common reason was a lack of guiding catheter support [30]. In this study, the most common reason for failure to advance the catheter into the ascending aorta was radial spasm. In contrast, in our study, the most common reason for the failure of transradial PCI was a lack of guiding catheter support. This may be explained by reduced radial spasm, the use of a single catheter, a more inferior take-off of the right coronary artery, and the presence of more calcified coronary lesions or long coronary artery stenoses.

Previous studies have limited data on the use of 0.038 wires in coronary angiography for the visualization of the right coronary artery with a single catheter [20]. In our study, we used both the rigid tip and the soft tip of the 0.038-inch wire in more than half of the patients. No data are available on the use of the 0.014 wire with the 0.038 wire for catheter support in single-catheter studies. We used a 0.038 guidewire and a 0.014 floppy wire together in 16 patients. All of these techniques not only prevent the guiding catheter from being inadvertently positioned in the conus branch but also reduce the risk of coronary dissection.

An important consideration when interpreting our findings is the potential for selection bias. Patients in whom the procedure could not be completed with a single JL 3.5 guiding catheter had a higher prevalence of previous coronary artery disease and coronary artery anomalies, suggesting more complex coronary and vascular anatomy. Therefore, some of the observed advantages associated with the single-catheter strategy, including shorter procedure duration, lower contrast use, reduced fluoroscopy time, and lower radiation exposure, may partly reflect differences in anatomical complexity rather than the catheter strategy alone. Because of the observational and non-randomized nature of the study, causality cannot be established. Prospective randomized studies comparing single-catheter and conventional approaches in anatomically comparable patient populations are warranted.

## 5. Study Limitations

This study had a number of limitations. First, the open-label nature of this trial could have introduced potential bias in the outcomes. Second, the study’s primary focus was on evaluating the feasibility of the JL 3.5 guiding catheter for primary PCI in STEMI patients, with no control group included. Third, a comparison could not be made with other catheters, such as the Ikari Left 3.5 guiding catheter (Terumo, Tokyo, Japan), which is more commonly used for single-catheter coronary intervention. Fourth, all procedures were performed by operators with substantial experience in transradial coronary interventions. Therefore, the high procedural success rate observed in this study may not be directly reproducible in lower-volume centers or among operators with less experience in radial techniques. Further multicenter studies involving different levels of operator expertise are needed to assess the generalizability of these findings. Fifth, since the right radial artery is the default access point, the manipulation methods for the JL3.5 guiding catheter may not be suitable for left radial access. Sixth, the study was susceptible to subjectivity due to variations in operators’ practices, which could impact procedure time and contrast volume; nonetheless, this aligns with real-world scenarios.

## 6. Conclusions

In the absence of specialized multipurpose catheters, the JL 3.5 guiding catheter can be a reliable and efficient option for performing both right and left coronary interventions via the right radial approach. This single-catheter technique demonstrates high success in cannulation of both arteries and contributes to reduced procedure time, contrast volume, fluoroscopy duration, door-to-balloon and needle-to-balloon times, radiation dose, radial access complications, and procedural costs. It is particularly advantageous in STEMI cases, where time efficiency is critical. Further evidence from prospective randomized controlled studies is needed to support these observations.

## Figures and Tables

**Table 1:** Baseline clinical and demographic characteristics.

	All Patients (*N* = 1139)	Single Catheter (*n* = 1038)	Without Single Catheter (*n* = 101)	*p*
Age, years	60 ± 11	59.6 ± 11.5	62 ± 12.8	0.046
Male gender, *n* (%)	909 (79.8)	836 (80.5)	73 (72.3)	0.048
BMI, kg/m^2^	28 ± 4.6	28.3 ± 4.6	28.9 ± 4.6	0.213
Diabetes mellitus, *n* (%)	319 (28.0)	288 (27.7)	31 (30.7)	0.529
Hypertension, *n* (%)	468 (41.1)	426 (41.0)	42 (41.6)	0.916
Smoking, *n* (%)	753 (66.1)	693 (66.8)	60 (59.4)	0.319
Hyperlipidaemia, *n* (%)	79 (6.9)	70 (6.7)	9 (8.9)	0.413
History of CAD, *n* (%)	192 (16.9)	162 (15.6)	30 (29.7)	<0.001
History of stroke, *n* (%)	22 (1.9)	22 (2.1)	0 (0)	0.140
Familial CAD, *n* (%)	198 (17.4)	180 (17.3)	18 (17.8)	0.903
Systolic blood pressure, mmHg	126 ± 23	126.5 ± 23.8	127.6 ± 24.5	0.663
Diastolic blood pressure, mmHg	75 ± 11	74.5 ± 11.6	75.4 ± 11.8	0.893
Heart rate, bpm	76 (66–85)	76.5 (67–85)	76 (62–86)	0.932
MI type, *n* (%)				0.016
Anterior	412 (36.2)	386 (37.2)	26 (25.7)	
Inferior	645 (56.6)	578 (55.7)	67 (66.3)	
Isolated posterior	47 (4.1)	45 (4.3)	2 (2.0)	
Lateral	33 (2.9)	28 (2.7)	5 (5.0)	
Isolated right ventricular	2 (0.2)	1 (0.1)	1 (1.0)	
Killip status, *n* (%)				0.047
I	1095 (96.1)	997 (96.1)	98 (97.0)	
≥II	44 (3.9)	41 (3.9)	3 (3.0)	

BMI: Body mass index. CAD: Coronary artery disease. MI: Myocardial infarction.

**Table 2:** Procedural features and outcome parameters of the study population.

Variables	All Patients	Single Catheter	Without Single Catheter	*p*
Number of punctures	1.3 ± 0.7	1.3 ± 0.6	1.4 ± 0.7	0.091
Puncture time, s	17.6 ± 14.7	17.5 ± 14.5	18.5 ± 17.1	0.554
Number of catheters, *n* (%)				<0.001
1	1039 (91.2)	1038 (100)	0 (0)	
2	87 (7.6)	0 (0)	87 (86.1)	
>2	14 (1.2)	0 (0)	14 (13.9)	
Use of wire, *n* (%)				<0.001
None	482 (42.3)	446 (43.0)	36 (35.6)	
Soft tip of the 0.038	341 (29.9)	313 (30.2)	28 (27.7)	
Rigid tip of the 0.038 wire	276 (24.2)	264 (25.4)	12 (11.9)	
Rigid tip of the 0.038 wire + 0.014 wire	16 (1.4)	15 (1.4)	1 (1.0)	
Stent/balloon, *n* (%)				0.011
DES	1067 (93.7)	980 (94.4)	87 (86.1)	
BMS	8 (0.7)	7 (0.7)	1 (1.0)	
Balloon	59 (5.2)	47 (4.5)	12 (11.9)	
DES + BMS	5 (0.4)	4 (0.4)	1 (1.0)	
Total contrast volume, mL	200 (150–250)	200 (150–250)	250 (200–320)	<0.001
Total procedure time, min	20 (15–29)	20 (15–26)	30 (23.6–40)	<0.001
Total fluoroscopy time, min	11 (7.6–15.6)	10.3 (7.3–14.8)	16.7 (12.6–24.4)	<0.001
Door-to-balloon time, min	15 (12–16)	15 (12–16)	15 (15–18)	<0.001
Needle-to-balloon time, min	6 (5–8)	6 (5–8)	9 (6.5–10)	<0.001
Total radiation dosage (mgray)	370 (213–627)	358 (210–604)	545 (330–820)	<0.001
Balloon pre-dilatation, *n* (%)	1081 (94.9)	985 (94.9)	96 (95)	0.946
Balloon post-dilatation, *n* (%)	634 (55.7)	581 (56)	53 (52.5)	0.499
Use of thrombus aspiration, *n* (%)	42 (3.7)	37 (3.6)	5 (5.0)	0.480
Number of stents, *n* (%)	1.2 ± 0.6	1.2 ± 0.6	1.3 ± 0.9	0.044
Culprit vessel, *n* (%)				0.001
LMCA	4 (0.4)	2 (0.2)	2 (2.0)	
LAD	434 (38.1)	409 (39.4)	25 (24.8)	
LCX	213 (18.7)	192 (18.5)	21 (20.8)	
RCA	475 (41.7)	422 (40.7)	53 (52.5)	
RI	13 (1.1)	13 (1.3)	0 (0)	
Number of diseased vessels, *n* (%)	1.4 ± 0.6	1.4 ± 0.6	1.4 ± 0.6	0.886
Coronary artery anomaly, *n* (%)	8 (0.7)	4 (0.4)	4 (4.0)	0.003
Femoral crossover, *n* (%)	24 (2.1)	0 (0)	24 (23.8)	<0.001
Access-site composite outcomes, *n* (%)	101 (8.9)	85 (8.2)	16 (15.8)	0.010
Access-site complications, *n* (%)				0.001
Spasm	54 (4.8)	41 (4.0)	13 (12.9)	
Occlusion	27 (2.4)	25 (2.4)	2 (2.0)	
Hematoma	16 (1.4)	16 (1.5)	0 (0)	
Perforation	4 (0.4)	3 (0.3)	1 (1.0)	
Iatrogenic coronary dissection, *n* (%)	3 (0.3)	1 (0.1)	2 (2.0)	0.022
30-day reinfarction, *n* (%)	20 (1.8)	19 (1.8)	1 (1.0)	0.458
30-day stroke, *n* (%)	2 (0.2)	2 (0.2)	0 (0)	0.830
30-day mortality, *n* (%)	21 (1.8)	19 (1.8)	2 (2.0)	0.569

BMS: Bare-metal stent. DES: Drug-eluting stent. LMCA: Left main coronary artery. LAD: Left anterior descending artery. LCX: Left circumflex artery. RCA: Right coronary artery. RI: Ramus intermedius artery.

**Table 3:** Procedural complications and short-term clinical outcomes according to sex.

Variable	All Patients (*n* = 1139)	Male (*n* = 909)	Female (*n* = 230)	*p* Value
Femoral crossover, *n* (%)	24 (2.1)	14 (1.5)	10 (4.3)	0.017
Access-site composite outcomes, *n* (%)	101 (8.9)	59 (6.5)	42 (18.3)	<0.001
Access-site complications, *n* (%)				
Spasm	54 (4.8)	37 (4.1)	17 (7.4)	0.028
Occlusion	27 (2.4)	14 (1.5)	13 (5.7)	<0.001
Hematoma	16 (1.4)	9 (1.0)	7 (3.0)	0.040
Perforation	4 (0.4)	0 (0.0)	4 (1.7)	0.002
Iatrogenic coronary dissection, *n* (%)	3 (0.3)	3 (0.3)	0 (0.0)	1.000
30-day stroke, *n* (%)	2 (0.2)	2 (0.2)	0 (0.0)	1.000
30-day mortality, *n* (%)	21 (1.8)	12 (1.3)	9 (3.9)	0.019

**Table 4:** Univariate and multivariate logistic regression analyses for predictors of successful single-catheter procedural completion in STEMI patients.

	Univariate Logistic Regression				Multivariate Logistic Regression			
	ODDS Rate	95% Confidence Interval		*p*	ODDS Rate	95% Confidence Interval		*p*
Age	1.01	1.00	1.03	0.047				
Male gender	1.58	1.00	2.52	0.050	1.55	0.95	2.54	0.081
History of CAD	2.28	1.44	3.61	<0.001	0.44	0.27	0.70	0.001
Coronary anomaly	0.09	0.02	0.37	<0.001	0.08	0.02	0.35	0.001

CAD: Coronary artery disease.

**Table 5:** Mechanisms and causes of failure of transradial percutaneous coronary intervention performed with a single catheter.

**Failure of Arterial Access**	
Inadequate arterial puncture	2
**Failure to Advance Catheter into Ascending Aorta**	
Radial artery spasm	11
Radial artery loop/tortuosity	4
Take-off angle between the ascending aorta and the brachiocephalic artery <90° or type I aortic arch	3
High take-off of the radial artery	2
Radial artery dissection/rupture	1
**Failure to Complete Percutaneous Coronary Intervention due to Lack of Guiding Catheter Support**	
Inferior right coronary artery take-off	29
Calcific coronary lesion or long coronary artery stenosis	21
Take-off angle between the left main coronary artery and the left circumflex artery <90°	10
Subclavian tortuosity	7
Ascending aortic dilatation	5
Horizontal aorta	4
Coronary artery anomaly	2

## Data Availability

The data presented in this study are not publicly available due to patient privacy and ethical restrictions but may be available from the corresponding author upon reasonable request.
